# Prognosis and distribution of ischemic stroke with negative diffusion-weighted imaging: a systematic review and meta-analysis

**DOI:** 10.3389/fneur.2024.1376439

**Published:** 2024-04-26

**Authors:** Ahmed Alkhiri, Fahad Alturki, Nayef M. Alansari, Ahmed A. Almaghrabi, Basil A. Alghamdi, Aser F. Alamri, Saeed Alghamdi, Seraj Makkawi

**Affiliations:** ^1^College of Medicine, King Saud Bin Abdulaziz University for Health Sciences, Jeddah, Saudi Arabia; ^2^King Abdullah International Medical Research Center, Jeddah, Saudi Arabia; ^3^College of Medicine, King Saud Bin Abdulaziz University for Health Sciences, Riyadh, Saudi Arabia; ^4^King Abdullah International Medical Research Center, Riyadh, Saudi Arabia; ^5^Neuroscience Department, King Faisal Specialist Hospital and Research Center, Jeddah, Saudi Arabia; ^6^Department of Neuroscience, Ministry of the National Guard-Health Affairs, Jeddah, Saudi Arabia

**Keywords:** ischemic stroke, diffusion-weighted imaging, false-negative, meta-analysis, intracerebral hemorrhage

## Abstract

**Background:**

Magnetic resonance diffusion-weighted imaging (DWI) is the most sensitive modality for ischemic stroke diagnosis. However, DWI may fail to detect ischemic lesions in a proportion of patients.

**Methods:**

Following PRISMA statement, a systematic search of Medline, Embase, and Web of Science was conducted until January 3, 2024. The inclusion was confined to English literature with sufficient reporting. Proportions of DWI-negative ischemic stroke were pooled. For binary variables, odds ratios (ORs) were computed using the random-effects model.

**Results:**

Fourteen studies constituting 16,268 patients with a clinical diagnosis of ischemic stroke and available DWI findings were included. Intravenous thrombolysis (IVT) was administered to 19.6% of the DWI-negative group and 15.3% of the DWI-positive group. DWI-negative ischemic stroke was reported in 16% (95% CI: 10–24%; after sensitivity analysis: 11% [95% CI: 8–15%]) of stroke patients. Among minor stroke patients (National Institutes of Health Stroke scale [NIHSS] of 5 or less), 24% (95% CI 12–42%) had negative DWI findings. Predictors of DWI-negative scans included posterior circulation stroke, history of ischemic heart disease, prior stroke, or prior transient ischemic attack. Cardioembolic stroke (OR, 0.62, 95% CI: 0.41–0.93) and history of atrial fibrillation increased the likelihood of positive DWI findings (OR, 0.56, 95% CI: 0.45–0.71). Patients with DWI-negative ischemic stroke had higher odds of good functional outcomes (modified Rankin scale [mRS] of 0–1) (OR, 2.26; 95% CI: 1.03–4.92), lower odds of stroke recurrence (OR, 0.68; 95% CI: 0.48–0.96), and lower odds of severe disability or mortality (mRS of 3–6) (OR, 0.44; 95% CI: 0.34–0.57) compared to patients with positive DWI. Rates of symptomatic intracerebral hemorrhage after IVT were comparable between groups.

**Conclusion:**

DWI-negative findings were present in a significant proportion of ischemic stroke patients and may be utilized as a marker for favorable prognosis.

## 1 Introduction

Ischemic stroke is a highly debilitating condition that contributes significantly to long-term disability and mortality (1). Magnetic resonance imaging (MRI) studies play a pivotal role in the diagnosis of cerebral infarctions (2). Magnetic resonance diffusion-weighted imaging (DWI) represents the gold-standard imaging modality of cerebral ischemia diagnosis with a sensitivity of 88–100% and a specificity of 95–100% (3). Moreover, DWI studies can be utilized to guide the use of reperfusion therapy (4). Yet, DWI studies are not perfect and can miss ischemic lesions in a proportion of stroke patients. Prior studies suggested that 6.8% of ischemic stroke patients may have negative DWI studies (5). This might be more pronounced in milder stroke cases, as previous studies suggested that one-third of minor stroke individuals may exhibit negative DWI findings (6).

Ultimately, stroke diagnosis depends on components of clinical reasoning in the context of proper clinical history and physical examination. This comes with particular importance in patients with neutral MRI findings (7). Notably, physicians may undermine the diagnosis of stroke in patients exhibiting negative DWI, and therefore important treatment decisions such as thrombolytic treatment and proper secondary preventive strategies can be undermined (8). Prior large prospective studies identified the presence of ischemic lesion as a clear marker of higher risk of stroke recurrences after transient ischemic attacks (TIAs), especially in high-risk cases (9, 10). Moreover, the shift from a time-based definition to a tissue-based definition has been motivated mainly by the evidence that virtually all TIA/stroke cases have a subtle central nervous system damage which can be shown with biomarkers even more sensitive than DWI (11, 12). Previous reports have discussed the prevalence of DWI-negative ischemic stroke, which occurred more in patients with posterior circulation strokes, hyperacute presentations, and small infarct volumes (3, 5). However, long-term outcomes of ischemic stroke patients with negative DWI findings remain unrevealed. Hence, we aim to assess the distribution and the prognostic value of negative DWI findings in patients with clinical diagnosis of ischemic stroke.

## 2 Methods

This review was reported in compliance with the Preferred Reporting Items for Systematic Reviews and Meta-Analyses (13). This review was conducted according to a prespecified registered protocol (PROSPERO: CRD42024497583). Neither patient consent nor institutional approval was required for the current study as this work constitutes aggregate data meta-analysis of previously published studies.

### 2.1 Search method and resources

We systematically searched three electronic databases (Medline, Embase, and Web of Science) until January 3, 2024. To ensure coverage of wider literature, a hands-on search of grey literature and reference lists of retrieved full-texts was also conducted. The search strategy was tailored to each database using combinations of relevant terms. The full search strategy is provided in the Supplementary material.

### 2.2 Study selection process

Independent reviewers performed title and abstract screening, which was followed by a full-text assessment. The inclusion criteria of the current review were as follows (1): confirmed clinical diagnosis of ischemic stroke and (2) sufficient reporting, which entailed adequate reporting of essential study characteristics (such as explicit stroke definition and the provision of separate data for stroke and TIA to allow independent extraction of stroke data) as well as adequate data on patient characteristics for each group (patients with negative AND positive DWI scans). Studies of transient ischemic attacks, abstract conferences, and reviews were excluded. Studies fulfilling the inclusion criteria were selected for final analysis.

### 2.3 Data extraction and risk of bias

Two reviewers completed the data extraction process. Extracted data included study characteristics, patients’ demographics, medical history details (including comorbidities and smoking history), and short and long-term outcomes.

Independent reviewers assessed the quality of included studies using the Newcastle-Ottawa scale (NOS) for observational studies (14). Conflicts in the assessment were resolved through discussion with a third author. Scoring stars of 7 to 9, 5 or 6, and 0 to 4 indicate good, moderate, and poor quality, respectively. Conflicts were resolved through consultation with a senior reviewer.

### 2.4 Statistical analysis

Data analyses were conducted using RevMan and R software. A *p*-value of less than 0.05 was determined for statistical significance. Data were pooled using a random-effects model. The generalized linear mixed model (GLMM) was adopted to pool the prevalence of DWI-negative ischemic stroke. In a prespecified sensitivity analysis, we excluded studies that only included patients with minor stroke. In addition, proportions of DWI-negative among patients with minor stroke [defined as National Institutes of Health Stroke scale (NIHSS) less than or equal to 5] and posterior circulation were pooled separately. Odds ratios (ORs) and their corresponding 95% confidence intervals (CIs) were computed for dichotomous variables. We performed multiple subgroup analyses based on stroke severity and based on follow-up duration (discharge, 90-days, and 1-year). The Higgins index (I^2^) was used to measure heterogeneity, where I^2^ values greater than 50% were regarded as significant.

## 3 Results

### 3.1 Search results

**Figure 1:**
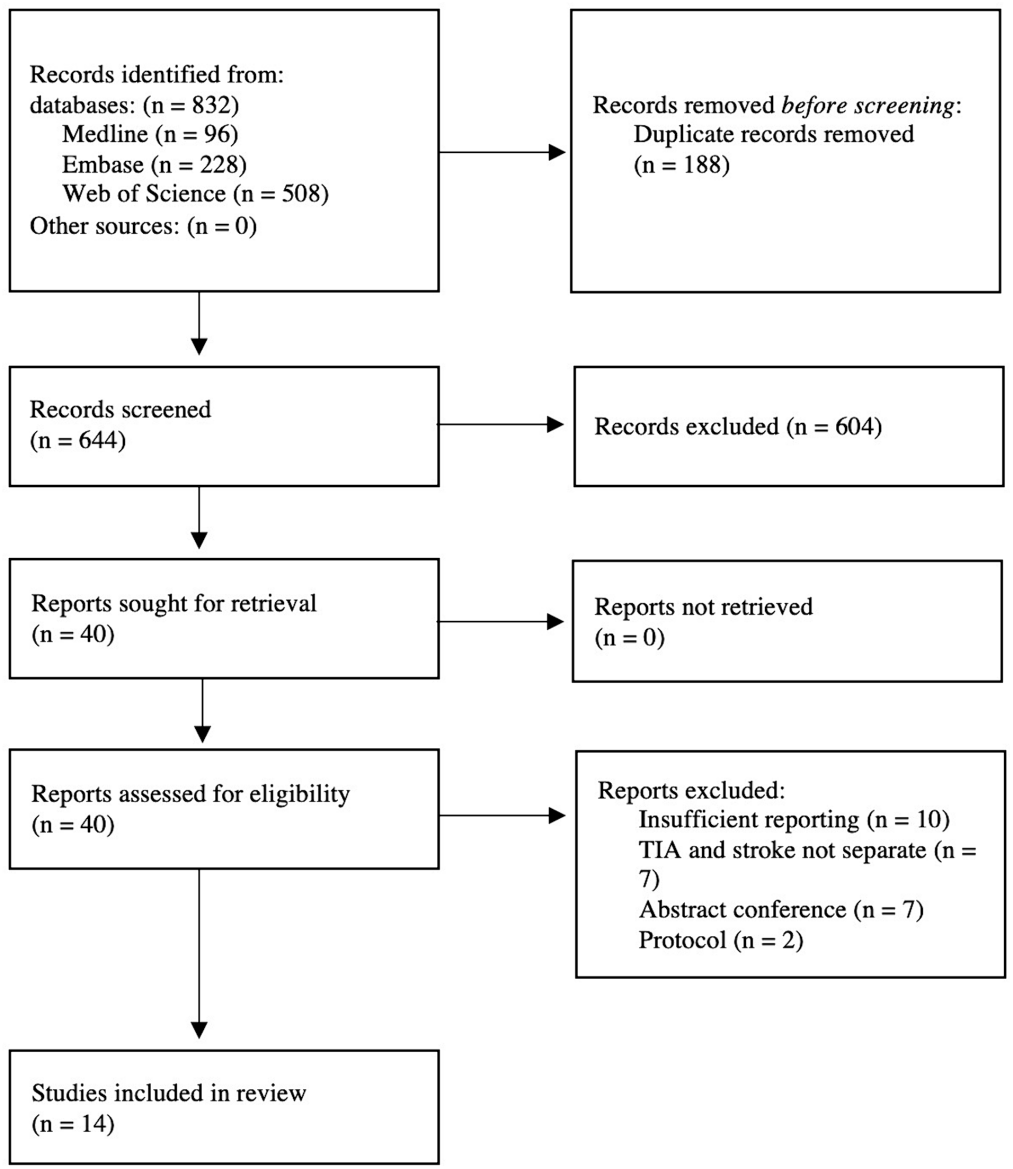
Flow diagram detailing the literature search process.

We identified 832 citations through the database search. Of those, 188 were duplicates and subsequently excluded. Next, 604 references were excluded through titles and abstracts screening. We retrieved 46 potentially eligible studies for full-text screening, of those, 14 eligible studies satisfied our inclusion criteria (Figure 1).

### 3.2 Study and patient characteristics

Fourteen studies (2, 3, 6–8, 15–23) satisfied our inclusion criteria. The included studies were published between 2000 and 2023. We included 16,268 patients with clinical diagnosis of ischemic stroke and with available MRI-DWI findings. A total of 2,563 patients (15.8%) received intravenous thrombolysis (IVT), with 350 (19.6%) in the DWI-negative group and 2,213 (15.3%) in the DWI-positive group. Among the included studies that reported time from stroke onset to DWI scan, there was variability in this interval. Doubal et al. (6) reported a median of 12 days. Six other studies reported data comparing time to scan between DWI-negative and DWI-positive groups. The median time ranged from 2 h (17) to 6 days (8) for DWI-negative patients, compared to 1.81 h (17) to 4 days (8) for DWI-positive patients. Details of age, gender, and stroke severity distribution are summarized in Table 1.

**Table 1:** Characteristics of included studies.

Study	Stroke cases, *N*	DWI-ve, *N* (%)	Mean age	% male	Median baseline NIHSS	Time from stroke onset to DWI scan	Intravenous thrombolysis, *N* (%)	Criteria of stroke diagnosis	NOS
Doubal et al. (6)	246	81 (33)	Total: 68.1	Total: 66	Total: 2	Total: 12 (4–27) (days)^*^	NR	Clinical Dx at discharge, f/u MRI	9
Hurford et al. (15)	400	240 (60)	DWI-ve: 66; DWI+ve: 70^*^	DWI-ve: 49.6; DWI+ve: 55.7	DWI-ve: 1; DWI+ve: 1	DWI-ve: 5 (12); DWI+ve: 3 (3) (days)*	NR	Clinical Dx at discharge, f/u CT or MRI	8
Makin et al. (8)	264	76 (29)	DWI-ve: 66; DWI+ve: 67^*^	DWI-ve: 49; DWI+ve: 62	DWI-ve: 2; DWI+ve: 2	DWI-ve: 6 (3–11); DWI+ve: 4 (2–9) (days)^*^	NR	Clinical Dx at discharge, f/u MRI	9
Zuo et al. (2)	349	33 (9.46)	DWI-ve: 66.97; DWI+ve: 68.26	DWI-ve: 45.5; DWI+ve: 63.6	DWI-ve: 2.82; DWI+ve: 3.74^§^	NR	NR	Clinical Dx at discharge, f/u CT or MRI	8
Yaghi et al. (7)	709	199 (28)	Total: 63.5	Total: 49	DWI-ve: 1; DWI+ve: 2	NR	NR	Clinical Dx at discharge, f/u MRI	9
Wang et al. (3)	12,026	932 (7.7)	DWI-ve: 63; DWI+ve: 63^*^	DWI-ve: 59.3; DWI+ve: 69.3	DWI-ve: 2; DWI+ve: 4	DWI-ve: 2 (1–4); DWI+ve: 2 (1–4) (days)^*^	DWI-ve: 152 (16.3); DWI+ve: 978 (8.8)	Clinical Dx at discharge, f/u CT or MRI	8
Oppenheim et al. (16)	139	8 (5.8)	Total: 58	Total: 87.5	NR	DWI-ve: 8 (8.5) (hours)^§^	NR	Clinical Dx at discharge, f/u MRI	6
Simonsen et al. (17)	565	47 (8.3)	DWI-ve-ve: 62; DWI+ve: 67	DWI-ve: 59.6; DWI+ve 62.2	DWI-ve: 4; DWI+ve: 7	DWI-ve: 2 (1.64–2.57); DWI+ve: 1.81 (1.33–2.33) (hours)^*^	DWI-ve: 47 (100); DWI+ve: 518 (100)	Clinical Dx at discharge, f/u MRI	7
Brunser et al. (18)	711	90 (13.7)	DWI-ve: 67.6; DWI+ve: 69.8	NR	DWI-ve: 2; DWI+ve: 4	DWI-ve: 8.54 (16.98); DWI+ve: 18.83 (30.81) (hours)^§^	DWI-ve: 39 (43.3); DWI+ve: 152 (24.5)	Clinical Dx at discharge, f/u CT or MRI	8
Li et al. (19)	437	54 (12.36)	DWI-ve: 62.1; DWI+ve: 62.5	DWI-ve: 68.5; DWI+ve: 73.9	DWI-ve: 2.5; DWI+ve: 5	NR	DWI-ve: 54 (100); DWI+ve: 383 (100)	Clinical Dx at discharge, f/u MRI	7
Bulut et al. (20)	116	11 (9.48)	Total: 71.5	Total: 51.7	DWI-ve: 5.0, DWI+ve: 6.17^§^	DWI-ve: 4.3 (1.2); DWI+ve: 10.79 (10.26) (hours)^§^	NR	Clinical Dx at discharge	7
Nisar et al. (21)	66	3 (4.54)	Total 53.8	DWI-ve: 33.3; DWI+ve: 57.1	NR	NR	NR	Clinical Dx at discharge	6
Giraldo et al. (22)	89	23 (26)	DWI-ve: 52; DWI+ve: 62	DWI-ve: 45; DWI+ve: 55	DWI-ve: 6; DWI+ve: 11	NR	DWI-ve: 23 (100); DWI+ve: 66 (100)	Clinical Dx at discharge, f/u CT and MRI	6
Zhu et al. (23)	151	35 (23.2)	DWI-ve: 64.7; DWI+ve: 64.8	DWI-ve: 62.9; DWI+ve: 69	DWI-ve: 3; DWI+ve: 4	NR	DWI-ve: 35 (100); DWI+ve: 116 (100)	Clinical Dx at discharge, f/u CT or MRI	6

§ Data presented as mean. DWI indicates diffusion-weighted imaging; NR, not reported; MRI, magnetic resonance imaging; CT, computed tomography; NOS, Newcastle-Ottawa scale; Dx, diagnosis. * Data presented as median.

For quality assessment of the included studies, four studies were deemed to be of moderate quality, while the remaining articles were judged to be of good quality according to the NOS (Table 1).

### 3.3 Prevalence

**Figure 2:**
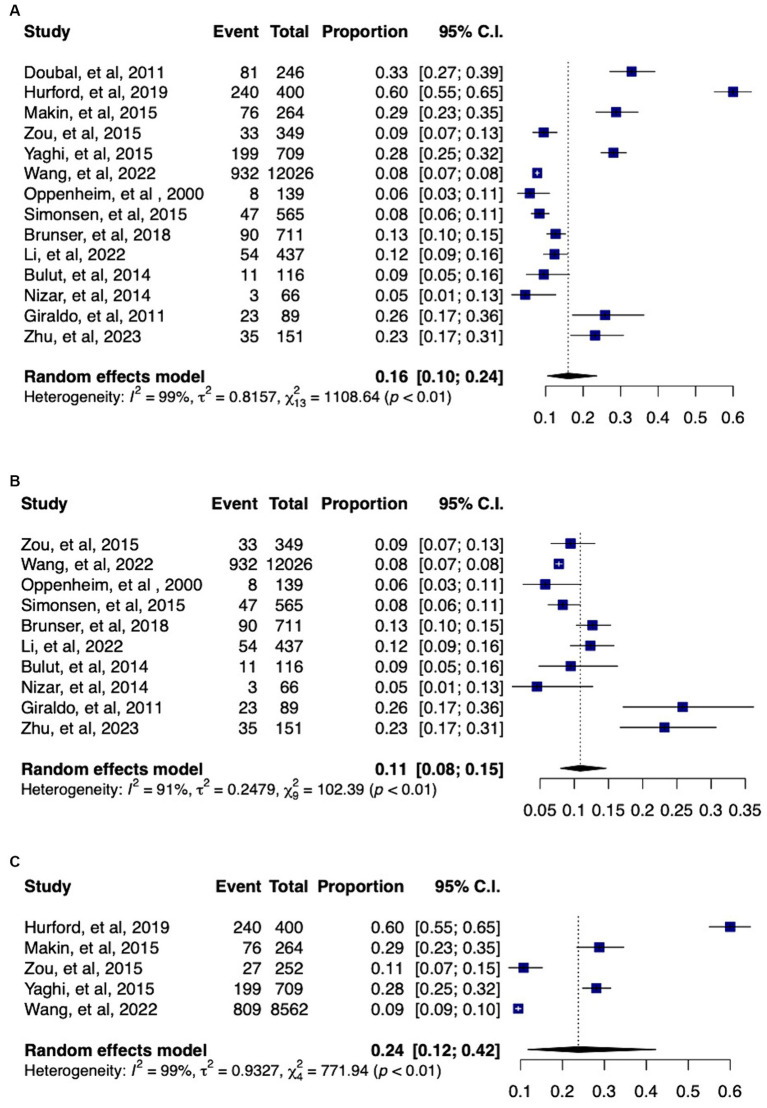
Forest plots of the pooled proportions of DWI-negative ischemic stroke **(A)** from all 14 included studies **(B)** after sensitivity analysis and removal of studies only reporting on minor ischemic stroke (NIHSS ≤5) **(C)** from studies reporting only on minor stroke patients.

DWI-negative ischemic stroke was found in 16% (95% CI: 10–24%) of stroke patients, with significant heterogeneity (I^2^ = 99%). After conducting sensitivity analysis and removal of studies reporting only on minor stroke individuals (NIHSS of less than or equal to 5), the prevalence of DWI-negative stroke was further refined to 11% (95% CI: 8–15%) with significant between-study heterogeneity (I^2^ = 91%). Among patients with minor ischemic stroke, the prevalence of DWI-negative scans was 24% (95% CI: 12–42%), with significant heterogeneity (I^2^ = 99%) (Figure 2) The prevalence of DWI-negative scans in cases of anterior circulation ischemic stroke was found to be 7% (95% CI: 4–14%; I^2^ = 83%). Posterior circulation ischemic stroke demonstrated a higher occurrence of negative scans, with a prevalence of 19% (95% CI: 16–22%) and no between-study heterogeneity (I^2^ = 0%) (Supplementary Figure S1).

### 3.4 Predictors

Among elements of medical history prior to index stroke, history of ischemic heart disease, prior history of stroke, and prior history of TIA were all found to be significant predictors of DWI-negative stroke. In terms of etiology classifications, there was a significantly lower likelihood of DWI-negative findings in cardioembolic stroke cases (OR, 0.62, 95% CI: 0.41–0.93). However, no statistically significant differences were observed in patients with large artery atherosclerosis (OR, 0.68, 95% CI: 0.45–1.03) or small artery occlusion (OR, 0.71, 95% CI: 0.05–9.32). Posterior circulation stroke resulted in significantly higher odds of DWI -ve lesions compared to anterior circulation stroke (OR, 2.47, 95% CI: 1.30–4.72). On the contrary, patients with atrial fibrillation were more likely to experience DWI-positive stroke (OR, 0.56, 95% CI: 0.45–0.71). No significant difference was observed between the status of DWI scans and other parameters such as female gender, history of smoking, diabetes, hypertension, and dyslipidemia (Table 2).

**Table 2:** Elements or predictors of DWI scan status among ischemic stroke patients.

Elements or predictors	No. studies	No. patients	Odds ratio (IV, random, 95% CI)
Female	4	1,691	1.16 (0.64, 2.10)
Posterior circulation stroke^§^	6	1702	2.47 (1.30, 4.72)*
Large artery atherosclerosis	5	14,088	0.68 (0.45, 1.03)
Small artery occlusion	5	14,088	0.71 (0.05, 9.32)
Cardioembolism	5	14,088	0.62 (0.41, 0.93)*
Prior stroke	5	13,165	1.42 (1.03, 1.96)*
Prior transient ischemic attack	2	12,290	1.96 (1.39, 2.77)*
Atrial fibrillation	8	15,031	0.56 (0.45, 0.71)*
Diabetes mellitus	9	15,054	0.92 (0.80, 1.06)
Hypertension	9	14,992	0.98 (0.83, 1.16)
Ischemic heart disease	4	12,878	1.36 (1.13, 1.64)*
Dyslipidemia	7	14,554	1.14 (0.97, 1.34)
Smoking	9	14,992	0.81 (0.63, 1.04)

* Indicates *p* value < 0.05. ^§^ Odds of DWI -ve scans only of posterior circulation stroke versus anterior circulation stroke. mRS indicates modified Rankin Scale; sICH, symptomatic intracerebral hemorrhage IV, inverse variance; CI, confidence interval.

### 3.5 Outcomes

We pooled aggregate data to demonstrate the prognosis of DWI-negative ischemic stroke. Table 2 demonstrates the pooled analysis of stroke outcomes. In short, rates of stroke recurrence were lower in patients with initial negative DWI scan (OR, 0.68, 95% CI: 0.48–0.96). A similar effect was reported across subgroup analyses based on follow-up duration at 90-days and among minor stroke patients. However, this effect was not sustained at 1-year follow-up (OR, 0.77; 95% CI: 0.38–1.55). Moreover, the odds of good functional outcomes (defined as a modified Rankin Scale [mRS] of 0 or 1) were higher in patients with DWI-negative ischemic stroke (OR, 2.26; 95% CI: 1.03–4.92). In the subgroup analyses, this effect was sustained at hospital discharge and 90-days follow-up but not after 1-year of index stroke. Rates of severe disability or mortality (defined as mRS of 3–6) were significantly lower in patients with DWI-negative ischemic stroke (OR, 0.44; 95% CI: 0.34–0.57), with sustained effect in favor of DWI-negative stroke (compared to DWI-positive) at discharge and 1-year follow-up. Rates of symptomatic intracerebral hemorrhage (sICH) were substantially lower among patients with negative DWI scans after IVT (OR, 0.22 95% CI: 0.04–1.14) (Table 3).

**Table 3:** Stroke outcomes of patients with DWI-negative ischemic stroke compared to DWI-positive.

Outcome or subgroup		No. studies	No. patients	Odds ratio (IV, random, 95% CI)
Stroke recurrence		3	12,678	0.68 (0.48, 0.96)*
	In minor stroke patients^§^	3	9,214	0.65 (0.43, 0.96)*
	At 90-days in minor stroke patients	1	388	0.50 (0.24, 1.05)
	At 1-year in minor stroke patients	2	8,826	0.77 (0.38, 1.55)
Good functional outcomes (mRS 0–1)		3	1,266	2.26 (1.03, 4.92)*
	At discharge	1	437	4.03 (2.12, 7.65)*
	At 90-days	1	565	2.61 (1.24, 5.52)*
	At 1-year	1	264	1.16 (0.68, 1.99)
Severe disability or mortality (mRS 3–6)		2	12,177	0.44 (0.34, 0.57)*
	At discharge	1	151	0.06 (0.01, 0.48)*
	At 1-year	1	12,026	0.46 (0.35, 0.59)*
sICH post intravenous thrombolysis		2	426	0.22 (0.04, 1.14)

* Indicates *p* value < 0.05. ^§^ Minor stroke was defined as an NIH stroke scale (NIHSS) of 5 or less. mRS indicates modified Rankin Scale; sICH, symptomatic intracerebral hemorrhage; IV, inverse variance; CI, confidence interval.

## 4 Discussion

The current systematic review and meta-analysis findings indicate that a notable proportion of ischemic stroke patients present with DWI-negative scans, particularly in the context of minor stroke. Among variables of medical history and vascular risk factors prior to index stroke, clinical history of stroke, TIA, or ischemic heart disease can increase the odds of negative DWI findings. Negative scans were more common in posterior circulation stroke. In contrast, cardioembolic stroke and history of atrial fibrillation were associated with more positive scans. In addition, a favorable profile was reported in patients with DWI-negative ischemic stroke with more improvement in good functional outcomes, fewer stroke recurrences, and lower odds of disability and mortality.

DWI imaging plays a fundamental diagnostic role in the workup of select acute ischemic stroke patients who might benefit from reperfusion therapies, including IVT and mechanical thrombectomy (24). The aim of the current study was not to question the pivotal role of DWI imaging in the settings of ischemic stroke. Rather, this investigation aimed to increase the awareness of DWI-negative ischemic stroke as a potential clinical manifestation. In this review, we found that 11–16% of ischemic stroke patients may present with negative findings in the DWI sequence. We reported a slightly higher prevalence of negative DWI findings in routine stroke patients, including minor and non-minor stroke subsets. A previous meta-analysis suggested that 6.8% of ischemic stroke patients may have negative DWI scans (5). The difference in the percentages may stem from the variable proportion of minor stroke cases, geographical distribution, and statistical pooling approaches. Furthermore, the prevalence of DWI-negative events was more pronounced in milder stroke cases (24%). Possible explanations exist for the high prevalence of DWI-negative events in minor stroke. First, higher NIHSS scores may represent more extensive tissue infarction, detectable by DWI (25). Second, during the event of reduced cerebral blood flow, the degree of hypoperfusion in minor stroke causes symptoms but is insufficient to induce changes visible on DWI (26).

In addition, variables associated with DWI-negative ischemic stroke have not been evaluated in detail (18). Our pooled findings showed that among different aspects of medical history and vascular risk factors, prior history of stroke, TIA, or ischemic heart disease appeared to predispose towards DWI neutrality. In contrast, cardioembolic etiology and history of atrial fibrillation were more associated with positive lesions. Emboli from cardiac sources, including those raised from atrial fibrillation, can have varying sizes and often cause acute severe strokes contributing to higher rates of positive DWI lesions (27). Moreover, factors that have been associated with DWI-negative ischemic stroke in the literature included longer times from symptom onset to MRI, lower NIHSS scores, smaller posterior circulation lesions, as well as clinical presentations such as ataxic hemiparesis, internuclear ophthalmoplegia, and lateral medullary infarction syndromes (28). Our results provide further evidence supporting the association between posterior circulation ischemia and DWI -ve stroke (5, 16, 17). DWI abnormalities in posterior circulation tend to appear later in the acute phase compared to anterior circulation stroke (2). This comes with particular importance when there is a short time interval between stroke onset and imaging acquisition which may limit the ability of DWI to capture sufficient signals to detect posterior circulation lesions. In addition, brainstem lesions tend to be smaller and might be overlooked due to the presence of magnetic susceptibility artifacts (16, 20). In our analysis, posterior circulation ischemia was more associated with negative scans compared to anterior circulation stroke (19% vs. 7%; OR, 2.47, 95% CI: 1.30–4.72).

The prognostic implications of DWI-negative ischemic stroke have not been fully explored. Evidence from recent studies has demonstrated poorer prognostic outcomes associated with DWI-positive lesions (8, 29). However, no systematic synthesis has been obtained in this regard so far. In our study, DWI-negative ischemic stroke had a more favorable profile compared to DWI-positive events. These findings are in line with previous reports demonstrating that DWI-negative can be used as an imaging indicator of favorable prognosis. Yet, most included patients in our pooled analysis were derived from studies with major inclusion of minor stroke individuals. Therefore, our findings further corroborate Wang et al. (3) notion which suggests the potential value of negative DWI in the risk stratification of minor stroke individuals. However, limited data exists regarding the optimal treatment approach for these individuals. Within our study, IVT did not elevate the risk of sICH among patients with negative DWI. In the case of TIA patients, a recent report demonstrated that the benefits of dual antiplatelet therapy (DAPT) were restricted to those with positive DWI (30). Nevertheless, there is a scarcity of efficacy data comparing IVT, DAPT, and single antiplatelet therapy (SAPT) in the context of DWI-negative stroke. Currently, international guidelines (24, 31) recommend stratifying individuals with minor strokes based on disability level to guide the administration of IVT. IVT is recommended in minor disabling stroke. In contrast, no additional benefits of IVT were observed in non-disabling stroke (32), and a dual antiplatelet regimen may offer similar efficacy with a superior safety profile (33). Incorporating DWI status into decision-making process and its role in guiding acute and secondary prevention strategies for stroke patients warrants further investigation. Noteworthy, the pooling of outcome data might be limited by the small number of contributing studies, and further evidence in this regard is needed.

Despite the clinical implications and strength of our review, certain limitations need acknowledgment. First, aggregate data were pooled rather than individual patient data. Second, the observational nature of the included studies may introduce bias to the overall synthesis. Third, we included studies published in varying periods, and technical evolutions of MRI machines may enable more detection of ischemic lesions. Fourth, median baseline NIHSS scores of most included studies were consistent with minor and moderate stroke subtypes, which have more proclivity of DWI-negative findings and could affect our results. However, mild stroke represents a substantial proportion of routine stroke patients presenting to the emergency room, which adds to the value of our study (34). Sixth, strict inclusion criteria limited the analysis of some variables. Lastly, our study is limited by intrinsic methodological differences and potential biases within the included studies, including the lack of reported follow-up computed tomography (CT) and/or MRI findings in two studies (20, 21). This raises concerns about the potential inclusion of patients with stroke mimics in the analysis.

## 5 Conclusion

In conclusion, this meta-analysis revealed that DWI-negative ischemic stroke represents a prevalent phenomenon, particularly among minor stroke patients. Additionally, DWI-negative scan may serve as an imaging marker for a favorable prognosis.

## Data availability statement

The original contributions presented in the study are included in the article/Supplementary materials, further inquiries can be directed to the corresponding author.

## Author contributions

AhA: Conceptualization, Data curation, Formal analysis, Funding acquisition, Investigation, Methodology, Project administration, Resources, Software, Supervision, Validation, Visualization, Writing – original draft, Writing – review & editing. FA: Writing – original draft, Writing – review & editing. NA: Writing – original draft, Writing – review & editing. AAA: Writing – original draft, Writing – review & editing. BA: Writing – original draft, Writing – review & editing. AFA: Writing – original draft, Writing – review & editing. SA: Writing – review & editing. SM: Writing – original draft, Writing – review & editing.
