# Supplementary material for: Prognosis and distribution of ischemic stroke with negative diffusion-weighted imaging: a systematic review and meta-analysis
Source: Front Neurol. 2024 Apr 26;15:1376439. doi: 10.3389/fneur.2024.1376439 (PMC11082379; doi:10.3389/fneur.2024.1376439)
Supplement: Supplementary file 1 [file Data_Sheet_1.PDF]

Supplementary Material

1 Supplementary Table and Figures

1.1 Supplementary Table

Table S1. Complete search strategy.

Database	Searches	Results
Embase	('stroke'/exp OR 'stroke' OR 'cerebral infarction') AND ('negative diffusion weighted imaging'/exp OR 'negative DWI' OR 'DWI-negative'/exp)	228
WoS	(ALL=(Stroke) OR ALL=(Cerebral Infarction))<br>AND<br>(ALL=(negative diffusion weighted imaging) OR ALL=(negative DWI) OR ALL=(DWI-negative))	508
Medline	Ovid MEDLINE(R) ALL <1946 to January 14, 2022><br><br>1 exp Stroke/ 174984<br>2 stroke$.mp. 385637<br>3 cerebral infarction$.mp. 36046<br>4 negative diffusion weighted imaging$.mp. 29<br>5 negative DWI$.mp. 65<br>6 DWI-negative$.mp. 67<br>7 1 or 2 or 3 414421<br>8 4 or 5 or 6 134<br>9 7 and 8 96	96

1.2 Supplementary Figures

(A)

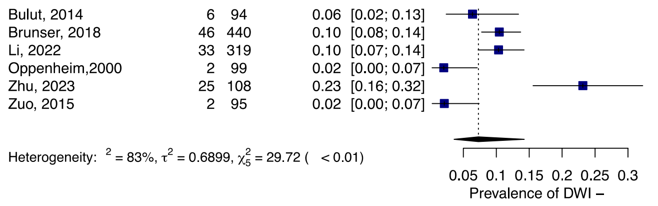

(B)

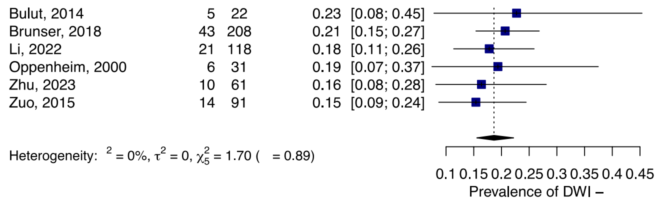

Supplementary Figure 1. Pooled proportions of DWI-negative ischemic stroke in the (A) anterior and (B) posterior circulation.
